# Role of DNA repair defects in predicting immunotherapy response

**DOI:** 10.1186/s40364-020-00202-7

**Published:** 2020-06-29

**Authors:** Jing Zhang, David J. H. Shih, Shiaw-Yih Lin

**Affiliations:** Department of Systems Biology, The University of Texas MD Anderson Cancer Center, Houston, TX 77030 USA

**Keywords:** DNA damage response, Immunotherapy, Biomarkers, Immune checkpoint blockade

## Abstract

Defect in DNA damage response (DDR) is a common feature of cancer cells, which regulates tumor growth and therapeutic response. Recently, the approval of immune checkpoint blockade (ICB) for tumors with defective mismatch repair has paved the way for investigating the role of other DDR defects in sensitizing cancer to ICB therapy. Despite great progress in understanding DDR pathways, the mechanisms that link DDR defects and ICB response remain incompletely understood. Further, the clinical activity of ICB in patients with DDR defective tumors has not been well described. Here, we discuss recent studies demonstrating that biomarkers in DDR pathways may serve as potential predictors to guide the selection of patients for ICB therapy. A better understanding of the relationship between deficiency in DDR and response to ICB would facilitate efforts in optimizing the efficacy of immunotherapy.

## Introduction

Immune checkpoint blockade (ICB), using antibodies against inhibitory signaling molecules expressed on tumor and immune cells, has shown unprecedented clinical activity across many tumor types. Currently, the approved use of anti-CTLA-4 (ipilimumab), anti-PD-1 (nivolumab and pembrolizumab), anti-PD-L1 (atezolizumab, avelumab and durvalumab), and their combinations have demonstrated significant improvements over chemotherapy in cancer patients [1, 2]. Despite this success, the majority of patients fail to respond to these ICB therapy due to the innate and acquired resistance [3].

Optimal design of ICB treatments will require multiple reliable predictive biomarkers that can help to select, before the initiation of treatment, patients who are most likely to benefit. For instance, Hodi et al. led a phase 3 ipilimumab study on patients with metastatic melanoma. They found that 20% of patients with metastatic melanoma achieved long-term benefit when treated with ipilimumab, 60% of patients had response for less than 2 years, and the rest showed no clinical benefit [4]. If we could predict, before ipilimumab treatment, whether a patient will be a long-term responder, short-term responder, or non-responder, we would be able to achieve the best therapeutic outcomes for the responders without causing unnecessary harm to non-responders. In a separate trial, Hodi et al. reported that 64% of patients treated with the nivolumab plus ipilimumab combination were still alive after 2 years [5]. However, this improvement in overall survival with the combination treatment came at significant cost: 54% of patients in the combination arm showed grade 3–4 adverse events, which is significantly higher than the ipilimumab-only arm (20%). Indeed, biomarkers would be critical to guide the selection of patients for increasingly efficacious yet also increasingly toxic ICB treatments. Currently, there are few biomarkers that effectively predict tumor response to ICB, so the search continues for such biomarkers. A promising area to conduct this search is at the interplay between cancer defects in DNA repair and the anti-cancer immune response.

**Fig. 1:**
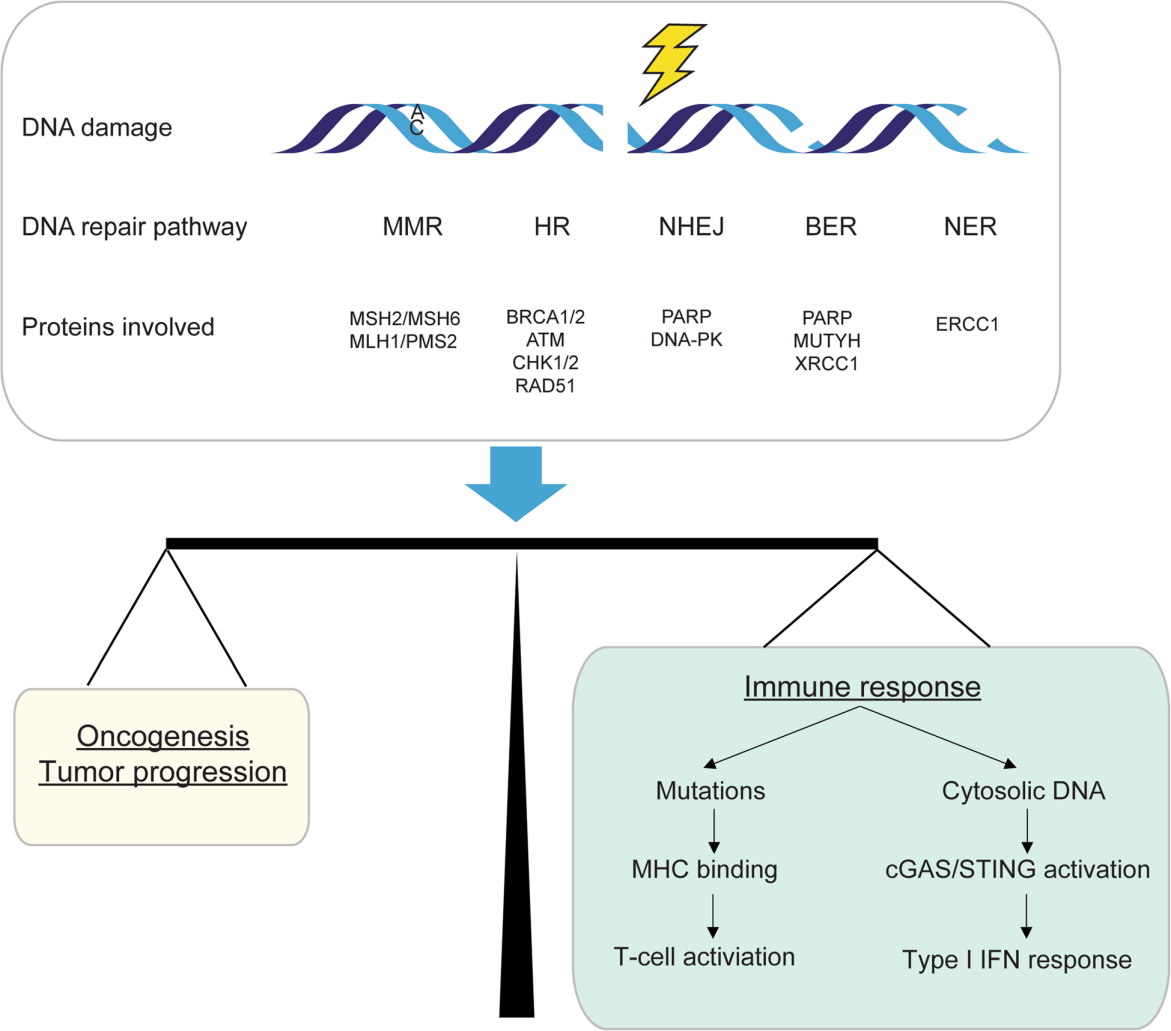
DNA damage response regulates tumor immunity. Defects in DNA damage response can result in both immunostimulation and immunosuppression. Production of neoantigens and/or activation of the cGAS/STING pathway can initiate anti-tumor immunity

In order to preserve the integrity of their genomes, cells initiate DNA damage response (DDR) pathways for different types of DNA damage. Based on the DNA lesion, DDR comprises 5 major pathways, including mismatch repair (MMR), homology-dependent recombination (HR), non-homologous end joining (NHEJ), base excision repair (BER), and nucleotide excision repair (NER) [6] (Fig. 1). Defects in DDR pathways result in a variety of genomic aberrations and accelerate tumor development [7]. However, in addition to promoting tumorigenesis, DDR defects in many cancers also provide therapeutic opportunities to kill cancer cells without affecting normal cells. An successful example of targeting DDR defects is the use of poly(ADP ribose) polymerase (PARP) inhibitors for the treatment of HR defective cancers [8–11]. When the activity of PARP is inhibited, normal cells survive owing to their ability to repair damaged DNA via the HR pathway. In contrast, cancer cells with HR defect caused, for example, by mutations in *BRCA1/2*, *BRD4*, *PTEN* or other HR related genes are sensitive to PARP inhibitors [12, 13]. Mounting evidence indicates that DDR defects are also important in driving sensitivity and response to ICB. To date, microsatellite instability/defective mismatch repair (MSI/dMMR) is a validated DDR defect biomarker for predicting response to ICB therapy that is approved by FDA [14]. The potential for defects in other DDR pathways to serve as predictive biomarkers for ICB response is less well investigated. In this review, we summarize the emerging evidence that elucidates the relationship between DDR pathways and ICB response, and we also discuss the promising roles of DNA repair proteins as predictive biomarkers to guide the use of ICB therapy.

## Existing predictive biomarkers for ICB in solid tumors

### PD-L1 as a predictive biomarker

Due to the importance of PD-L1 pathway in cancer development, PD-L1 (also known as CD274 or B7-H1) expression is one of the earliest and most promising predictive biomarkers. The initial phase 1 study in 2012 assessed the antitumor activity of nivolumab in patients with various advanced solid tumors [15]. Their study suggested that PD-L1 expression in tumors may predict clinical outcomes to anti-PD-1 therapy across many tumor types. Similar findings were reported by Taube et al. in 2014 who found that the pre-treatment level of PD-L1 expression is correlated with response to anti-PD-1 blockade [16]. Interestingly, PD-L1 expression by immune cells but not by tumor cells can also predicts the response to ICB across multiple cancer types [17, 18].

However, evidence from other studies shows that a substantial portion (20–30%) of PD-L1 negative patients respond to anti-PD-1 therapy [19, 20], which weakens the use of only PD-L1 status for predicting response. In addition, multiple other studies did not find that PD-L1 positive patients benefit from anti-PD-1 therapy [21, 22], which could be explained by the complexity of PD-L1 biology. PD-L1 expression is inducible and changes dynamically [23, 24], and PD-L1 is stored in intracellular reserves: tumor cells may translocate intracellular PD-L1 to the cell surface following the clearance of anti-PD-L1 antibodies [25]. Additionally, there is no standardized criteria and cutoff threshold for assessing PD-L1 expression level, which compromises the accurate evaluation of PD-L1 status [26, 27]. These studies collectively highlight important limitations of using PD-L1 as a sole predictive biomarker for ICB treatment.

### Tumor mutation burden and other potential biomarkers

Tumor mutation burden (TMB) is another promising predictive biomarker of ICB response. It measures the total number of tumor mutations, often within limited genomic regions, using high-throughput sequencing technologies [28]. The association of TMB with anti-PD-L1 or anti-CTLA4 therapy has been widely investigated. A clinical study in 2014 found that melanoma patients with higher pre-therapy TMB derived durable clinical benefit from anti-CTLA-4 treatment, though the authors noted that TMB alone was not sufficient to predict response accurately [29]. Indeed, this association between TMB and ICB was confirmed by another study of patients with melanoma [30]. By analyzing the sequencing data from pre-therapy tumors, the authors discovered that TMB and neoantigen load were significantly associated with clinical benefit from ICB. Similar findings linking TMB and ICB were observed in many other tumor types, including non-small-cell lung cancer (NSCLC) [21, 31], small cell lung cancer (SCLC) [32], and urothelial carcinoma [33, 34].

Despite the significant correlation between TMB and anti-PD-L1 therapy in various tumor types [28], many patients with high TMB do not respond to ICB and vice versa. For instance, patients with renal cell carcinoma [35], squamous cell carcinoma of the head and neck [36], breast or pancreatic cancer [37] show no significant association between TMB and response to ICB. Interestingly, a bioinformatic analysis of 68 patients with melanoma concluded that changes in TMB after 4 weeks of nivolumab treatment was strongly associated with anti-PD-1 response [38]. While this finding may be useful in early assessment of patient’s response to ICB, it would be challenging to implement it into clinical practice due to the requirement of on-therapy biopsies. In addition, the assays for measuring TMB, such as whole exome sequencing and targeted next generation sequencing [39], are expensive and complex. Clinical applications of these assays would require further standardization of these assays, including the determination of the optimal cutpoint for distinguishing tumors with high vs. low TMB.

With the improved understanding of tumor immune microenvironment, several promising candidates have emerged as predictive biomarkers. For example, the density of tumor-infiltrating lymphocytes (TILs) within the tumor may predict response to anti-PD1 therapy [40, 41]. Furthermore, *STK11/LKB1* mutations in lung adenocarcinoma was correlated with primary resistance to anti-PD1 therapy [42]. Interestingly, in a study of 35 patients with metastatic clear cell renal cell carcinoma, patients who have *PBRM1* loss can benefit from anti-PD-L1 therapy [43], and the authors validated this association in a follow-up study [44]. However, McDermott et al. reported that *PBRM1* mutations were not associated with anti-PD-L1 therapy based on the results from a phase 2 study in 305 patients with renal cell carcinoma [35]. The author instead showed that that blocking VEGF may sensitize patients to ICB treatment. Therefore, the biology of PBRM1 and ICB response is likely complex and remains largely unexplored. In patients with melanoma or uterine leiomyosarcoma, loss of *PTEN* is associated with inferior outcomes with anti-PD1 therapy [45, 46]. Additionally, recent studies suggested that *EGFR* or *ALK* mutant patients with NSCLC do not respond to pembrolizumab irrespective of PD-L1 expression [47–49].

Above all, there is a growing number of studies identifying predictive biomarkers for ICB response. These studies collectively suggest that, owing to the complexity of tumor-immune dynamics, single biomarkers are insufficient to accurately predict clinical outcomes with ICB, and optimal prediction of ICB response will require the identification of additional predictor biomarkers. Due to the interaction between DDR pathways and immune response, studying how DDR pathways influence antitumor immunity may provide fruitful insights, as well as revealing biomarkers of DDR deficiency that predict clinical response to ICB.

## The association between DDR defects and immunotherapy response

### MSI/dMMR

To date, the strongest evidence for the relationship between DDR defects and response to ICB therapy involves the DNA mismatch repair pathway. MSI together with dMMR are two broadly used predictive biomarkers [50]. MMR pathway is an essential DNA repair mechanism that identifies and repairs mismatched bases during DNA replication and genetic recombination [51]. dMMR, due to mutations or promoter methylation of any of four key genes—*MLH1*, *MSH2*, *MSH6* and *PMS2*—leads to the accumulation of mismatch errors and results in MSI and tumorigenesis [52]. Although MSI or dMMR rarely appears in breast cancer [53], prostate cancer [54] and lung adenocarcinoma [55], they are widely observed in other tumor types, including colorectal cancer [56, 57], gastric and gastrointestinal cancer [58, 59], and endometrial cancer [60].

Given that dMMR tumors harbor a large number of mutations, which are associated with high neoantigen load and T-cell infiltration [61, 62], it is not surprising that dMMR tumors can respond well to immune checkpoint blockade. A phase 2 clinical trial in 2015 investigated the activity of pembrolizumab in 41 patients who had either MMR-deficient or MMR-proficient metastatic carcinomas [63]. This study showed that patients with MMR-deficient tumors had higher response rates to anti-PD-L1 therapy than patient with MMR-proficient tumors. In an expanded study across 12 different tumor types, the authors found that around 20% of patients had achieved complete response, confirming that dMMR is a useful biomarker for predicting the response to ICB [64]. Based on these findings, the FDA, for the first time in its history, approved the use of pembrolizumab for the treatment of tumors that are indicated solely by biomarkers, irrespective of tumor site [65].

Although MSI/dMMR can serve as effective biomarkers to predict ICB response, some tumors with MSI high display resistance to ICB treatment, which challenges the idea that MSI/dMMR can be used as the sole biomarker in all tumor types [66, 67]. Therefore, identifying other DDR related biomarkers would be a promising avenue for predicting clinical response to ICB treatment.

### HR defects

Among these widely studied DNA repair pathways, HR is an accurate and error-free pathway for repairing double strand breaks. Accumulating evidence points to the potential association between HR defect and ICB response. In high grade serous ovarian cancer, mutations in *BRCA1/BRCA2*, which are essential players in HR pathways, result in higher neoantigen load, increased TILs, as well as enhanced expressions of PD-1 and PD-L1 [68]. Similar findings were observed in patients with pancreatic ductal adenocarcinoma [69]. Elevated TILs have also been observed in *BRCA1/BRCA2* mutant prostate cancer [70, 71] and breast cancer [72, 73]. In contrast, a pooled analysis of five phase 2 studies showed that TIL density was not associated with HR defect or *BRCA1/2* mutation in early stage patients with triple negative breast cancer [74].

In addition, the use of ICB therapy in HR deficient cancers has showed conflictive results. By analyzing the whole exome sequences of 38 patients with melanoma previously treated with PD-1 inhibitors, Hugo et al. reported that ICB responders are enriched for *BRCA2* mutations [75]. Further, a patient with *BRCA2* mutant MSS prostate cancer was reported to be sensitive to ICB [71]. A phase 1b study of patients with recurrent or refractory ovarian cancer demonstrated that *BRCA* status was not associated with response to anti-PD-L1 treatment [76]. Together, these results indicate that whether HR defect increase susceptibility to ICB treatment remains to be determined.

### BER deficiency

DNA single strand breaks, which do not disrupt the helical structure of DNA, are detected and repaired through BER pathway [77]. BER defective tumors from patients with colon adenocarcinoma, breast invasive carcinoma, or uterine corpus endometrial carcinoma, exhibit increased neoantigen production and upregulated PD-L1 expression [78]. Prominent TIL has been observed in colorectal cancer patients with germline mutations in *MUTYH*, a key DNA glycosylase involved in BER [79, 80]. These studies hint that BER defective cancer may display increased susceptibility to ICB. However, clinical trials regarding the use of ICB in BER defective cancer have not been reported.

### NER defects

Bulky DNA adducts that significantly distort the DNA helix, caused by UV irradiation or alkylating agents, are repaired by NER pathway [81]. NSCLC patients with single nucleotide polymorphisms in the *ERCC1* gene (which encodes the XPF nuclease that incises the damaged segment of the DNA during NER) were more sensitive to anti-PD-L1 treatment [82]. In fact, the role of NER in sensitizing ICB therapy in other tumor types is less well characterized. Further studies should be performed to investigate whether biomarkers of NER defects could predict clinical benefit from ICB.

### POLD1/ POLE mutations

Additional potential markers of genomic instability and response to immunotherapy are mutations in polymerase delta 1 (*POLD1*) and polymerase epsilon (*POLE*). These proteins play important role in DDR and are essential for accurate DNA replication by proofreading. Similar to the phenotype of dMMR, *POLD1* or *POLE* mutant tumors displayed increased TMB, neoantigen load, TILs, and effector cytokine levels [83–86], indicating that these mutated tumors may respond to ICB therapy. Recently, Wang et al. analyzed *POLE/POLD1* mutations in 47,721 patients with different types of cancer and found that *POLE/POLD1* mutations are promising predictive biomarkers for ICB [87]. Ongoing ICB trials are enrolling patients with *POLE*-mutant tumors [88].

The relationships between DDR defects and ICB response are summarized in Table 1.

**Table 1:** Summary of the relationships between DDR defects and ICB response

DDR defects	Tumor types	Criteria	Immunologic features	ICB response
MSI/dMMR	Colon, endometrial, gastric, ovarian	Germline mutations in DNA mismatch repair genes: *MLH1*, *MSH2*, *MSH6* or *PMS2*	Increased TMB, neoantigen load, TILs, and expression of PD-1 or PD-L1	Patients with MMR-deficient tumors had improved response rate to anti-PD-1 therapy: NCT01876511
				Ongoing trials: NCT02563002, NCT02912572, NCT02899793, NCT03150706, NCT03435107
HR defects	Ovarian, breast, prostate, pancreatic	Germline mutations in *BRCA1* or *BRCA2*	Increased neoantigen load, TILs, and expression of PD-1 or PD-L1	ICB therapy in HR deficient cancers has shown conflicting results: NCT01772004, Refs. [71, 75]
				Ongoing trials: NCT03025035, NCT03428802, NCT02571725, NCT03101280, NCT02849496
*POLD1*/*POLE* mutations	Endometrial, colon,	Germline mutations in *POLD1* or *POLE*	Increased TMB, neoantigen load, TILs, and effector cytokine levels	Ongoing trials: NCT02912572, NCT02899793, NCT03150706, NCT03435107, NCT03428802
BER deficiency	Colon, breast, endometrial	Germline mutations in *MUTYH*	Increased neoantigen load and PD-L1 expression	Not reported
NER defects	Lung	Single nucleotide polymorphisms in *ERCC1*		Not reported

## Mechanisms underlying the effect of DDR deficiency in antitumor immunity

In the tumor microenvironment, defects in DDR can dramatically impact the balance between immune surviellence and tumor progression. Failed DNA damage repair results in the accumulation of genomic errors, which activates oncogenes and initiates tumor development [89, 90]. On the other hand, this genomic instability may trigger anti-tumor immune response through two major mechanisms (Fig. 1). The accumulation of mutations caused by defective DDR can encode tumor-specific neoantigens that are presented on the cell surface in the context of major histocompatibility complex class I. These neoantigens are, in turn, specifically recognized by T cells [91] and enhance the anti-tumor immune response. In support of the notion that DDR defects drive ICB response via the production of neoantigens, DDR mutations in gastroesophageal cancers are associated with high TMB and elevated PD-L1 expression [92]. In urothelial cancer, patients whose tumors harbored any mutations in DDR pathways showed better response to anti-PD-1/PD-L1 treatment [93].

However, numerous studies have showed that tumors with very few mutations can also be sensitive to ICB treatment [94]. Therefore, non-neoantigen-based mechanisms of DDR defects on ICB treatment have been proposed. Failure of DDR can result in the increase of cytosolic DNA, which binds to the cyclic GMP-AMP synthase (cGAS) and subsequently stimulates the innate immune response through STING pathway (Fig. 1) [95–98]. In contrast to DDR intact tumors, DDR deficient breast tumors had increased immune infiltration and elevated PD-L1 expression, due to cGAS/STING pathway activation instead of neoantigen production [99]. In cell-based assays, loss of *BRCA2* stimulated a cGAS/STING mediated interferon response [100]. Importantly, STING activation mediated by DNA-damaging agents is implicated in response to ICB therapy. Due to the important role of PARP inhibitors in inducing synthetic lethality in cancer cells with HR defect, multiple studies recently investigated the clinical activity of combination therapy with PARP inhibitors and anti PD-1/PD-L1 in different cancer types [101, 102]. Although this combination therapy showed promising anti-tumor activity, tumors from the responders with ovarian carcinoma are *BRCA1/2* wild-type or HR intact [103]. The observation that HR intact tumors may also respond to the PARP inhibitor and ICB combination could perhaps be explained by the the activation of the cGAS/STING [104]. Further, a preclinical study showed that STING-inducing CHK1 inhibitors may enhance efficacy of ICB treatment in patients with SCLC [105].

In addition to neoantigen production and cGAS/STING pathway, DDR deficiency may also increase tumor sensitivity by activating other signaling pathways. For example, inhibition of ATM has been identified to induce interferon-mediated innate immune response in a cGAS/STING independent, but TBK1- and SRC- dependent manner [106]. Furthermore, blocking Nedd8-mediated clearance pathway in an MSI and ICB-resistant syngeneic mouse model has been shown to improve the efficacy of anti-PD1 treatment [67]. Despite the extensive knowledge of DDR pathways, it remains largely unclear how DDR defects initiate anti-tumor immunity to enhance response to ICB.

## Conclusions and future directions

Although ICB induces robust anti-tumor immune responses across different cancer types, it remains challenging to identify reliable predictive biomarkers that guide patient selection for ICB treatment. Currently, only a few biomarkers are FDA-approved for clinical use, including PD-L1 expression and MSI/dMMR [1, 14]. Other emerging biomarker candidates, such as TMB, the density of TILs, and immunogenic neoantigen load, have shown their unique advantages as well as their own intrinsic limitations. Based on the current understanding of the clinical response to ICB, a single biomarker is not sufficient to predict who will likely benefit from ICB treatment.

In addition to the widely studied MSI/dMMR, defects in other major DDR pathways have shown to promote PD-L1 expression, elevate TILs, and increase neoantigen load, which are potential determinants of the response to ICB. Interestingly, patients with co-mutations in multiple DDR pathways exhibit favorable clinical response to ICB [107]. Therefore, it is important to better understand how biomarkers in various DDR pathways can serve as effective predictors of ICB response. Prospective pre-clinical and clinical studies are needed to fully understand the relationship between DDR defects and ICB response.

Because cancer cells are often defective in DDR and poorly tolerate further DNA damage, combination treatments of ICB immunotherapy with DNA damaging therapeutics may enhance therapy response. In fact, several ongoing trials are evaluating the efficacy of combining ICB therapy and PARP inhibitors [108]. Other inhibitors of DDR pathways, such as inhibitors of ATM, ATR, CHK1, CHK2, may also contribute to ICB combination. DDR deficiency plays a critical role in immunotherapy response, future research in this area would likely provide insightful clues for the finding the optimal multimodality combination therapy, as well as for discovering additional biomarkers that can collectively predict immunotherapy response and maximize clinical benefit.

## Data Availability

Not applicable.

## Abbreviations

DDR: DNA damage response
ICB: Immune checkpoint blockade
MMR: Mismatch repair
HR: Homology-dependent recombination
NHEJ: Non-homologous end joining
BER: Base excision repair
NER: Nucleotide excision repair
PARP: Poly(ADP ribose) polymerase
MSI: Microsatellite instability
dMMR: Defective mismatch repair
TMB: Tumor mutation burden
NSCLC: Non-small-cell lung cancer
SCLC: Small cell lung cancer
TILs: Tumor-infiltrating lymphocytes
